# Genetic and Environmental Risk Factors for Autism Spectrum Disorder in Saudi Arabia: A Systematic Review

**DOI:** 10.7759/cureus.94607

**Published:** 2025-10-14

**Authors:** Nazim F Hamed, Abdulrahman Mohammed Alqahtani, Faisal Alshaibani, Sakinah Mohammed Elsharif, Sadeem Ali Saeed Alamri, Ashraf Serhan

**Affiliations:** 1 Pediatrics, Security Force Hospital, Dammam, SAU; 2 Psychiatry, Security Force Hospital, Dammam, SAU; 3 Pediatric Neurology/Epileptology, King Fahd Hospital of the University (KFHU) Imam Abdulrahman Bin Faisal University (IAU), Dammam, SAU; 4 Medicine, College of Medicine and Surgery, Al Baha University, Al- Baha, SAU; 5 Pediatrics, Security Forces Hospital, Dammam, SAU

**Keywords:** autism spectrum disorder, consanguinity, environmental risk factors, genetic risk factors, saudi arabia, systematic review

## Abstract

Aim: This study aimed to systematically synthesize evidence on the genetic and environmental risk factors associated with autism spectrum disorder (ASD) in the Saudi population.

Background: ASD is a complex neurodevelopmental disorder with a strong etiological basis in genetic and environmental interactions. The high rate of consanguinity in Saudi Arabia may amplify the burden of recessive genetic variants, making the investigation of region-specific risk factors a critical public health priority.

Materials and methods: This systematic review was conducted according to the Preferred Reporting Items for Systematic Reviews and Meta-Analyses (PRISMA) guidelines. A comprehensive search across PubMed, Web of Science, Scopus, and ScienceDirect was performed to identify studies on ASD risk factors in Saudi Arabia. Two independent reviewers screened records, extracted data, and assessed the risk of bias using the Quality Assessment of Diagnostic Accuracy Studies-2 (QUADAS-2) tool and the Newcastle-Ottawa Scale (NOS).

Results: Thirteen studies met the inclusion criteria. Genetic analyses revealed significant risk associations with specific Y-chromosome haplotypes, NR4A2 loss-of-function variants, TBX1 copy number variations (CNVs), and MTHFR single-nucleotide polymorphisms (SNPs). Key environmental risk factors identified were prenatal phthalate exposure, maternal stress, vitamin D deficiency, and consanguinity. Several studies also implicated neuroinflammatory markers (e.g., PGE2, IFN-γ) and lipid metabolism dysregulation in the disorder's pathophysiology.

Conclusion: The etiology of ASD in Saudi Arabia is characterized by a complex interplay between genetic predisposition, often exacerbated by consanguinity, and prenatal environmental exposures. While these findings are consistent with global research, they highlight distinct regional patterns. Future investigations should employ larger, balanced cohorts and integrate epigenetic and prospective designs to facilitate the development of targeted preventive and therapeutic strategies.

## Introduction and background

Autism spectrum disorder (ASD) is a complex neurodevelopmental condition defined in modern diagnostic manuals by persistent impairments in two core domains: 1) social communication and social interaction, and 2) restricted, repetitive patterns of behavior, interests, or activities [1]. According to the Diagnostic and Statistical Manual of Mental Disorders, Fifth Edition (DSM-5) and the International Classification of Diseases, 11th Revision (ICD-11), these symptoms must be present from the early developmental period and cause significant functional limitations. The prevalence of ASD has risen significantly over the past two decades. Data from the Centers for Disease Control and Prevention (CDC) currently estimate that approximately one in 54 children is affected [2]. While the precise etiology remains incompletely understood, extensive research confirms that ASD arises from a complex interplay between genetic predispositions and environmental risk factors [3]. Genetic studies have identified numerous susceptibility loci, including copy number variations (CNVs), single-nucleotide polymorphisms (SNPs), and de novo mutations, which collectively contribute to neurodevelopmental alterations [4]. For instance, large-scale genomic analyses, such as those by the Autism Sequencing Consortium, have pinpointed over 100 risk genes, many of which are involved in synaptic function, neuronal signaling, and chromatin remodeling [5].

In parallel, environmental influences, such as prenatal exposures to toxins, maternal stress, nutritional deficiencies, and perinatal complications, have been increasingly recognized as modifiable risk factors for ASD [6]. Epidemiological studies suggest that endocrine-disrupting chemicals (e.g., phthalates, bisphenol A), maternal immune activation, and vitamin D deficiency may interact with genetic predispositions to elevate ASD risk [7]. Interestingly, ASD is more common in groups with high consanguinity rates, such as those in Saudi Arabia, most likely because of the increased transmission of recessive genetic variations [8]. Given that cultural and environmental exposures in Middle Eastern populations differ greatly from those in Western cohorts, this demographic context emphasizes the necessity for region-specific research to clarify the distinct genetic and environmental causes of ASD in these communities [9]. This systematic review attempts to summarize and assess the available data on the genetic and environmental variables that contribute to ASD in Saudi communities, given the growing incidence of ASD in Saudi Arabia and the significant knowledge gaps about its population-specific risk factors. 

## Review

Methods

This systematic review was conducted in accordance with the Preferred Reporting Items for Systematic Reviews and Meta-Analyses (PRISMA) guidelines [10] to ensure methodological rigor and transparency.

Search Strategy and Study Identification

A comprehensive search strategy was implemented across multiple electronic databases, including PubMed, Web of Science, Scopus, and ScienceDirect, to identify relevant studies examining the genetic and environmental risk factors associated with ASD in Saudi Arabia. The search utilized a combination of keywords and Medical Subject Headings (MeSH) terms related to "Autism Spectrum Disorder," "ASD," "genetics," "environmental exposure," "risk factors," "Saudi Arabia," and their respective variants. The initial database searches yielded a total of 412 records.

Screening and Eligibility Criteria

All identified records were imported into reference management software (Rayyan Qatar Computing Research Institute (QCRI), Hamad Bin Khalifa University (HBKU) in Doha, Qatar) [11] to organize the screening process. After the removal of 184 duplicate records, a total of 228 unique records were screened based on their titles and abstracts. This screening phase led to the exclusion of 125 records that did not meet the broad inclusion criteria. The full text of the remaining 103 reports was sought for retrieval; of these, 39 reports could not be retrieved, leaving 64 reports that were assessed for eligibility in full text.

Studies were included if they met the following criteria: (1) investigated genetic and/or environmental risk factors for ASD in Saudi populations, (2) included human participants of any age diagnosed with ASD or at risk of ASD, (3) were published in English or Arabic, and (4) provided original data (e.g., observational, case-control, cohort, or cross-sectional studies). Exclusion criteria comprised (1) studies not focused on Saudi populations, (2) review articles, editorials, case reports, or conference abstracts without primary data, (3) studies lacking clear methodology or outcomes related to ASD risk factors, and (4) duplicate publications.

Upon full-text assessment, 46 reports were excluded for the following reasons: 17 investigated wrong outcomes, 20 studied the wrong population, and 14 were excluded as they were conference abstracts without sufficient primary data. This process resulted in a final inclusion of 13 studies in the systematic review.

Data Extraction and Quality Assessment

To minimize bias, two independent reviewers performed the entire screening process, study selection, and data extraction. Discrepancies were resolved through discussion and consensus. Data were extracted using a standardized form, capturing key details such as study design, sample size, participant demographics, genetic markers, environmental exposures, and primary findings.

The methodological quality and risk of bias of the included studies were evaluated using the Newcastle-Ottawa Scale (NOS) [12] for observational studies and Quality Assessment of Diagnostic Accuracy Studies-2 (QUADAS-2) [13] for genetic association studies. Criteria for assessment included selection bias, comparability, exposure/outcome ascertainment, and statistical robustness. Studies were classified as having a low, moderate, or high risk of bias, and the findings were interpreted in light of these assessments.

Data Synthesis Strategy

Given the significant heterogeneity in study designs, methodologies, and reported outcomes, a qualitative synthesis approach was adopted. Key findings were summarized in evidence tables, categorizing studies by genetic and environmental risk factors. Subgroup analyses were conducted where feasible, focusing on specific genetic variants (e.g., NR4A2, MTHFR), environmental exposures (e.g., phthalates, prenatal stress), and demographic variables (e.g., consanguinity, gender differences). A meta-analysis was deemed inappropriate due to the variability in measurement methods and outcomes across the included studies.

**Figure 1:**
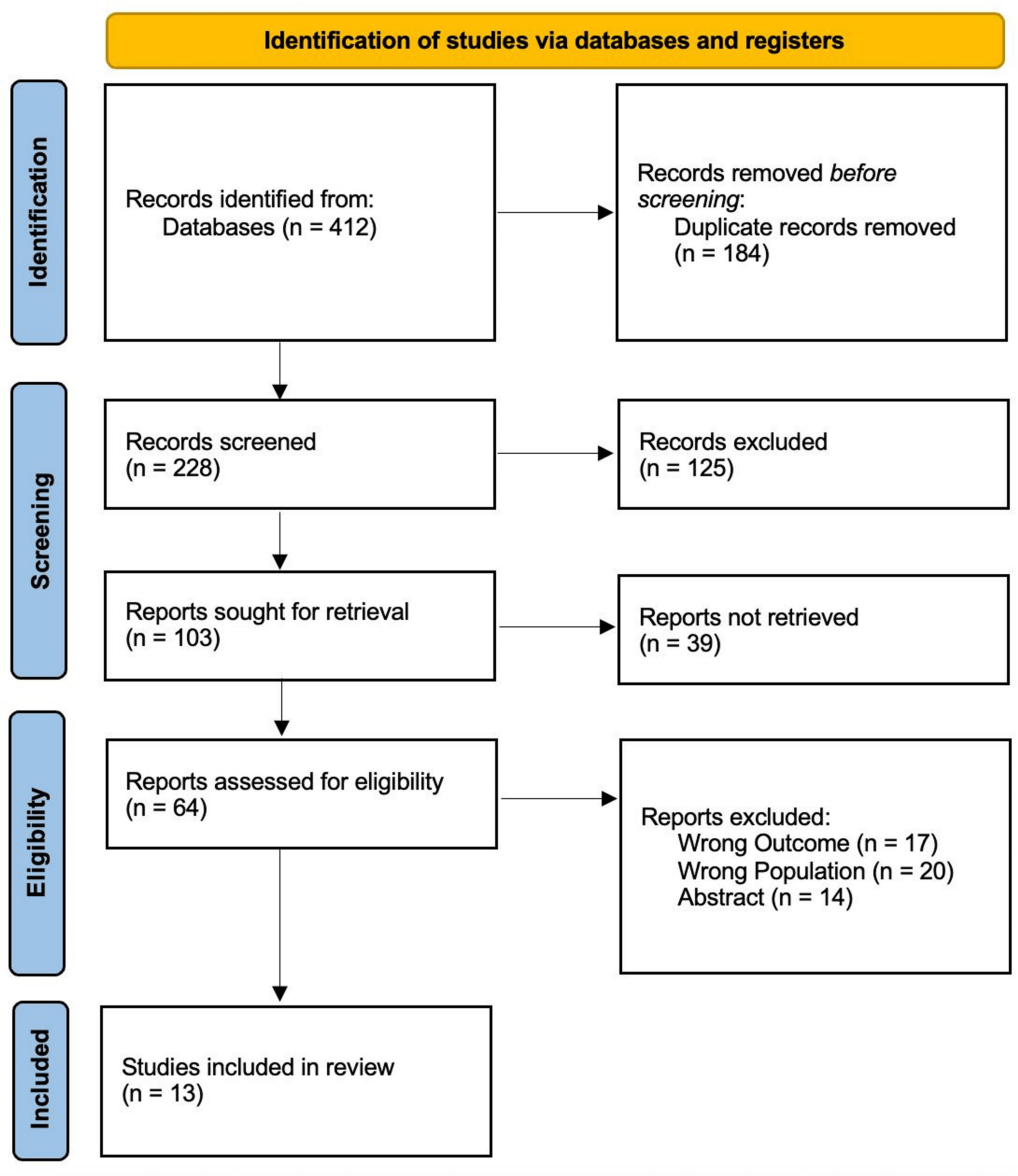
A PRISMA flowchart outlining the study selection process for the systematic review. PRISMA: Preferred Reporting Items for Systematic Reviews and Meta-Analyses

The study selection process is summarized in the PRISMA flow diagram (Figure 1).

Results

The demographic and methodological features of the 13 included studies that looked at environmental and genetic risk factors for ASD in Saudi Arabia are compiled in Table 1 [14- 26]. The studies varied in design, with most employing a case-control approach [15-17, 19-23, 25, 26], while others used cross-sectional [14], prospective cohort [21], or trio-based whole-exome sequencing (WES) [24] methodologies. Sample sizes ranged from small cohorts (e.g., eight cases in Bogari et al. [15]) to larger studies (e.g., 459 ASD cases in Alamoudi et al. [14]), with a notable focus on male participants in several studies [16, 25, 26] and one exclusively on females [19]. Geographic coverage included Makkah, Jeddah, Arar, and Dammam [14, 20], though many studies did not specify locations. Key demographic variables such as consanguinity, maternal age, and other studies, such as Oommen et al., emphasized prenatal exposures. [20], together with Al-Saleh et al. [21], highlighting how environmental factors and genetic predisposition interact in Saudi communities with ASD.

**Table 1:** Demographic and study characteristics ADHD: Attention-Deficit/Hyperactivity Disorder; CNV: Copy Number Variation; WES: Whole-Exome Sequencing; SNP: Single Nucleotide Polymorphism; MTHFR: Methylenetetrahydrofolate Reductase; PGE2: Prostaglandin E2; IFN-γ: Interferon-gamma; ROS: Reactive Oxygen Species; NM: Not Mentioned; M: Male; F: Female Ref.: Reference

Study (Author, Year) [Ref.]	Location in Saudi Arabia	Study Design	Sample Size (Cases/Controls)	Sample Type	Age Range (Years)	Gender (M/F)	Key Demographics
Alamoudi et al., 2023 [14]	Makkah & Jeddah	Cross-sectional	459 (cases only)	Children with ASD	2–14	NM	Mothers of ASD children; assessed prenatal stress
Bogari et al., 2020 [15]	NM	Case-control	8 (ADHD+ASD)/4 (ADHD only)	Pediatric patients	NM	NM	ADHD+ASD comorbidity focus
Alsubaie et al., 2020 [16]	NM	Case-control	47 (cases)/43 (controls)	Boys with ASD	NM (young boys)	Male only	Y-chromosome haplotypes
Alhazmi et al., 2022 [17]	NM	Case-control	15 (cases)/4 (controls)	Children with ASD	NM	NM	CNVs in chromosome 22
Alharbi et al., 2025 [18]	NM	Cohort (genetic)	338 (cases)/315 families	Children with ASD	NM	NM	NR4A2 variants
Almandil et al., 2023 [19]	NM	Case-control	22 (cases)/51 (controls)	Girls with ASD	NM	Female only	Olfactory receptor gene variants
Oommen et al., 2018 [20]	Arar & Dammam	Case-control	100 (cases)/100 (controls)	Children with ASD	3–10	NM	Environmental factors (consanguinity, diet)
Al-Saleh et al., 2021 [21]	NM	Prospective cohort	291 pregnant women	Maternal urine samples	NM (1st trimester)	NM	Phthalate exposure
Arab & Elhawary, 2019 [22]	NM	Case-control	112 (cases)/104 (controls)	Children with ASD	NM	NM	MTHFR gene variants
Elhawary et al., 2019 [23]	NM	Case-control	110 (cases)/102 (controls)	Children with ASD	NM	NM	Polygenic SNP interactions
Al-Mubarak et al., 2017 [24]	NM	Trio-based WES	19 trios (57 individuals)	Families with ASD	NM	NM	Inherited/de novo variants
El-Ansary & Al-Ayadhi, 2012 [25]	NM	Case-control	20 (cases)/19 (controls)	Children with ASD	NM	Male only	Neuroinflammatory markers
Qasem et al., 2018 [26]	NM	Case-control	47 (cases)/46 (controls)	Children with ASD	NM	Male only	Lipid metabolism markers

Table 2 synthesizes the key genetic and environmental risk factors identified across the studies. Genetic findings dominated, with multiple studies reporting Y-chromosome haplotypes [16], CNVs in chromosome 22 (e.g., TBX1) [17], and loss-of-function variants in NR4A2 [18] as significant contributors. Polygenic interactions involving HTR2A, SLC6A4, and BDNF SNPs [23] further underscored the complexity of ASD’s genetic architecture. Meanwhile, environmental factors such as prenatal phthalate exposure [21], maternal stress [14], and vitamin D deficiency [20] were linked to increased ASD risk. Notably, neuroinflammatory markers (PGE2, IFN-γ) [25,26] and lipid metabolism dysregulation [26] were proposed as biomarkers for ASD severity, bridging genetic susceptibility and environmental influences.

**Table 2:** Key variables and findings ADHD: Attention-Deficit/Hyperactivity Disorder; ASD: Autism Spectrum Disorder; AUTS2: Autism Susceptibility Gene 2; PCDH11Y: Protocadherin 11 Y-Linked; CNV: Copy Number Variation; TBX1: T-Box Transcription Factor 1; NR4A2: Nuclear Receptor Subfamily 4 Group A Member 2; LoF: Loss-of-Function; OR5V1: Olfactory Receptor Family 5 Subfamily V Member 1; OR12D2: Olfactory Receptor Family 12 Subfamily D Member 2; MTHFR: Methylenetetrahydrofolate Reductase; SNP: Single Nucleotide Polymorphism; HTR2A: 5-Hydroxytryptamine Receptor 2A; SLC6A4: Solute Carrier Family 6 Member 4; BDNF: Brain-Derived Neurotrophic Factor; USP9X: Ubiquitin Specific Peptidase 9 X-Linked; KDM5B: Lysine Demethylase 5B; SUMF1: Sulfatase Modifying Factor 1; WES: Whole-Exome Sequencing; PGE2: Prostaglandin E2; IFN-γ: Interferon Gamma; COX-2: Cyclooxygenase-2; NM: Not Mentioned; Ref.: Reference

Study (Author, Year) (Ref.)	Genetic Factors	Environmental Factors	Key Findings
Alamoudi et al., 2023 [14]	Family history of ASD	Prenatal stress	Family history was strongly associated with ASD severity; prenatal stress had a moderate effect.
Bogari et al., 2020 [15]	6 variants in ADHD+ASD patients	NM	Identified genetic overlap between ADHD and ASD.
Alsubaie et al., 2020 [16]	Y-haplotypes, AUTS2, PCDH11Y	NM	High-risk Y-haplotypes and 6 ASD-associated genes identified.
Alhazmi et al., 2022 [17]	TBX1 CNVs	NM	TBX1 deletions/duplications linked to ASD.
Alharbi et al., 2025 [18]	NR4A2 LoF variants	NM	Recurrent NR4A2 variants in Saudi ASD cases.
Almandil et al., 2023 [19]	OR5V1, OR12D2 variants	NM	Female-specific ASD biomarkers in olfactory receptor genes.
Oommen et al., 2018 [20]	NM	Consanguinity, maternal meds, vitamin D deficiency	Consanguinity and vitamin D deficiency are significant risk factors.
Al-Saleh et al., 2021 [21]	NM	Phthalate exposure (first trimester)	High phthalate levels linked to ASD risk.
Arab & Elhawary, 2019 [22]	MTHFR (677C>T, 1298A>C)	NM	MTHFR SNPs increase ASD susceptibility.
Elhawary et al., 2019 [23]	HTR2A, SLC6A4, BDNF SNPs	NM	Polygenic interactions contribute to ASD risk.
Al-Mubarak et al., 2017 [24]	USP9X, KDM5B, SUMF1 variants	NM	Novel and inherited variants in ASD trios.
El-Ansary & Al-Ayadhi, 2012 [25]	NM	Neuroinflammation (PGE2, IFN-γ)	Elevated inflammatory markers in ASD.
Qasem et al., 2018 [26]	NM	Lipid mediators (PGE2, COX-2)	PGE2 pathway dysregulation correlates with ASD severity.

Most studies demonstrated low-to-moderate risk, with Al-Mubarak et al. [24] and Alharbi et al. [18] scoring highest due to trio-based WES validation and clear phenotype-genotype correlations. However, Alamoudi et al. [14] and Oommen et al. [20] had moderate risk due to cross-sectional designs and potential recall bias in self-reported environmental exposures. El-Ansary & Al-Ayadhi [25] and Qasem et al. [26] showed low bias in biomarker quantification but lacked longitudinal validation.

Table 3 presents the risk of bias assessment for the 13 included studies, with the majority (nine studies) achieving an overall "low" risk of bias. Common sources of potential bias were related to the technical methods of exposure/outcome assessment (e.g., genotyping, biomarker assays) and statistical rigor, whereas most studies demonstrated low risk in participant selection and comparability.

**Table 3:** Risk of bias assessment NGS: Next-Generation Sequencing; WES: Whole-Exome Sequencing; aCGH: Array Comparative Genomic Hybridization; ES: Exome Sequencing; SNP: Single Nucleotide Polymorphism; CNV: Copy Number Variation; ELISA: Enzyme-Linked Immunosorbent Assay; n: sample size.

Study (Author, Year) [Ref.]	Selection Bias	Comparability	Exposure/Outcome Assessment	Statistical Rigor	Overall Risk
Alamoudi et al., 2023 [14]	Moderate	Moderate	Moderate (self-report)	High	Moderate
Bogari et al., 2020 [15]	Low	High (small n)	High (NGS validation)	Moderate	Moderate
Alsubaie et al., 2020 [16]	Low	Low	High (WES)	High	Low
Alhazmi et al., 2022 [17]	Moderate	Moderate	High (aCGH)	Moderate	Moderate
Alharbi et al., 2025 [18]	Low	Low	High (trio ES)	High	Low
Almandil et al., 2023 [19]	Low	Moderate	High (exome genotyping)	High	Low
Oommen et al., 2018 [20]	Moderate	Moderate	Moderate (questionnaire)	Moderate	Moderate
Al-Saleh et al., 2021 [21]	Low	Low	High (urinary biomarkers)	High	Low
Arab & Elhawary, 2019 [22]	Low	Low	High (TaqMan genotyping)	High	Low
Elhawary et al., 2019 [23]	Low	Low	High (SNPStats)	High	Low
Al-Mubarak et al., 2017 [24]	Low	Low	High (WES validation)	High	Low
El-Ansary & Al-Ayadhi, 2012 [25]	Low	Low	High (ELISA)	Moderate	Low
Qasem et al., 2018 [26]	Low	Low	High (lipid assays)	Moderate	Low

Discussion

The results of this systematic review support and build upon other studies that looked at environmental and genetic risk factors for ASD in Saudi Arabia and throughout the world. Our analysis identified strong genetic contributions, particularly Y-chromosome haplotypes and CNVs in chromosome 22 (e.g., TBX1), which corroborate earlier studies on male-biased ASD prevalence [16, 17]. These findings align with studies from throughout the world, including Sanders et al. (2015) [27], who emphasized how de novo mutations contribute to ASD, and Werling et al. (2016) [28], who emphasized sex-specific genetic architectures in neurodevelopmental disorders. The discovery of NR4A2 loss-of-function variants [18] further supports emerging evidence from Gandal et al. (2018) [29], who linked transcriptomic dysregulation in ASD to nuclear receptor genes. Additionally, the female-specific olfactory receptor gene variants (OR5V1, OR12D2) [19] provide novel insights into sex-differential ASD mechanisms, a finding that parallels recent work by Robinson et al. (2020) [30] on hormonally modulated genetic risk.

Environmental risk factors, particularly prenatal phthalate exposure [21] and maternal stress [14], were strongly associated with ASD in Saudi cohorts, reinforcing findings from Braun et al. (2014) [31], which linked endocrine-disrupting chemicals to neurodevelopmental impairments. The role of consanguinity and vitamin D deficiency [20] aligns with Al-Dbass et al. (2018) [32], who reported higher ASD prevalence in consanguineous Saudi families, and Vinkhuyzen et al. (2018) [33], who showed that a worldwide risk factor for ASD is a vitamin D deficit during pregnancy. Notably, PGE2 and IFN-γ are neuroinflammatory indicators [25, 26] observed in Saudi ASD cases that mirror Theoharides et al. (2016) [34], who proposed mast cell activation and neuroinflammation as key ASD etiological mechanisms. These findings collectively suggest that gene-environment interactions, particularly in high-consanguinity populations like Saudi Arabia, may exacerbate ASD susceptibility, as hypothesized by Hallmayer et al. (2011) [35].

While our review focused on Saudi populations, comparisons with global data reveal both commonalities and unique regional patterns. For instance, the high frequency of MTHFR variants [22] in Saudi ASD cases parallels meta-analyses by Guo et al. (2019) [36], which identified folate pathway disruptions as a transethnic ASD risk factor. However, the prevalence of Y-haplotype-driven ASD risk [16] appears more pronounced in Saudi males than in Western cohorts, possibly due to founder effects or consanguinity, a phenomenon also noted in Middle Eastern ASD studies by Aldosari et al. (2021) [37]. Conversely, the lack of robust epigenome-wide association studies (EWAS) in Saudi Arabia contrasts with advancements in European and North American research, such as Ladd-Acosta et al. (2020) [38], which identified DNA methylation signatures in ASD. This gap underscores the need for Saudi-specific epigenetic studies to clarify gene-environment interplay.

Strengths 

This review possesses several key strengths, including its rigorous adherence to the PRISMA guidelines and the use of standardized quality assessment tools like the QUADAS-2 and the NOS. Synthesizing evidence specifically from the understudied Saudi population, where high consanguinity rates create a unique genetic landscape, provides crucial region-specific insights into the interplay of genetic and environmental risk factors for ASD.

Limitations

Several limitations must be acknowledged. First, heterogeneity in study designs (e.g., cross-sectional vs. cohort) [14, 20, 21] limits direct comparability. Second, small sample sizes in genetic studies (e.g., Bogari et al. [15], n=8) reduce statistical power. Third, gender bias, with most studies focusing on males [16, 25, 26], may obscure female-specific ASD mechanisms. Fourth, retrospective environmental data (e.g., maternal stress recall [14]) raises the possibility of recollection bias. Lastly, unlike prospective birth cohort studies like the Norwegian Mother and Child Cohort, Saudi Arabia lacks longitudinal ASD cohorts, which limits the ability to draw conclusions about causality [39].

## Conclusions

This systematic review consolidates evidence for genetic (e.g., NR4A2, TBX1, MTHFR) and environmental (e.g., phthalates, consanguinity, vitamin D deficiency) risk factors in Saudi ASD populations, while highlighting neuroinflammation as a potential biomarker. The findings align with global research but also reveal region-specific patterns, likely influenced by consanguinity and unique environmental exposures. Future studies should prioritize larger, gender-balanced cohorts, epigenetic profiling, and prospective designs to establish causality. Public health initiatives targeting prenatal phthalate reduction and vitamin D supplementation could mitigate ASD risk in high-prevalence regions like Saudi Arabia.
